# Hereditary Trichilemmal Cysts are Caused by Two Hits to the Same Copy of the Phospholipase C Delta 1 Gene (*PLCD1*)

**DOI:** 10.1038/s41598-020-62959-z

**Published:** 2020-04-07

**Authors:** Michael S. Kolodney, Garrett C. Coman, Matthew B. Smolkin, Rachael Hagen, Jacob A. Katzman, Steven N Katzman, Alex C. Holliday, Joanna A. Kolodney

**Affiliations:** 1 Department of Dermatology, West Virginia University, Morgantown, WV USA; 2 Department of Dermatology, University of Utah, Salt Lake City, UT USA; 3 Department of Pathology, West Virginia University, Morgantown, WV USA; 4 Section of Dermatology, VA Tech School of Medicine, Roanoke, VA USA; 5 Department of Medicine, West Virginia University, Morgantown, WV USA

**Keywords:** Tumour-suppressor proteins, Molecular medicine

## Abstract

The autosomal dominant presentation of trichilemmal cysts is one of the most common single gene familial diseases in humans. However, the genetic basis for the inheritance and genesis of these lesions has remained unknown. We first studied patients with multiple trichilemmal cysts using exome and Sanger sequencing. Remarkably, 21 of 21 trichilemmal cysts from 16 subjects all harbored a somatic p.S745L (c.2234 G > A) mutation in phospholipase C delta 1 (*PLCD1*), a proposed tumor suppressor gene. In addition to this specific somatic mutation in their tumors, 16 of the 17 subjects with multiple trichilemmal cysts were also heterozygous for a p.S460L (c.1379 G > A) germline variant in *PLCD1* which is normally present in only about 6% of this population. The one patient of 17 that did not show the p.S460L germline variant had a germline p.E455K (c.1363 C > T) mutation in the same exon of *PLCD1*. Among 15 additional subjects, with a history suggesting a single sporadic trichilemmal cyst, six were likely familial due to the presence of the p.S460L germline variant. Of the remaining truly sporadic trichilemmal cysts that could be sequenced, only half showed the p.S745L somatic mutation in contrast to 100% of the familial cysts. Surprisingly, in contrast to Knudsen’s two hit hypothesis, the p.S745L somatic mutation was always on the same chromosome as the p.S460L germline variant. Our results indicate that familial trichilemmal cysts is an autosomal dominant tumor syndrome resulting from two hits to the same allele of *PLCD1* tumor suppressor gene. The c.1379 G > A base change and neighboring bases are consistent with a mutation caused by ultraviolet radiation. Our findings also indicate that approximately one-third of apparently sporadic trichilemmal cysts are actually familial with incomplete penetrance. Sequencing data suggests that the remaining, apparently sporadic, trichilemmal cysts are genetically distinct from familial cysts due to a lack of the germline mutations that underlie familial cysts and a decreased prevalence of the p.S745L somatic mutation relative to familial trichilemmal cysts.

## Introduction

**Figure 1:**
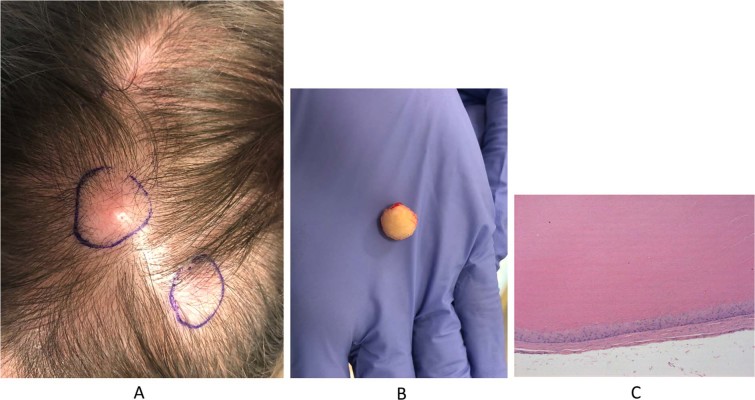
(**A**) Clinical presentation of representative subject. (**B**) Gross morphology of trichilemmal cyst. (**C**) Histology of representative trichilemmal cyst.

Trichilemmal cysts, also known as pilar cysts, are common cutaneous nodules that histologically resemble the hair follicle outer root sheath. These lesions are thought to occur in about five to ten percent of the US population^1^, and their removal is a common cutaneous surgical procedure. Trichilemmal cysts usually develop on the scalp, but can also present on the face, neck and extremities^2^. When excised, these lesions easily shell out as smooth, firm spheres consisting of a well-keratinized white lining enclosing soft keratinaceous material (Fig. 1). A trichilemmal cyst can occasionally transform into a rapidly proliferating trichilemmal tumor or trichilemmal carcinoma^3,4^.

Trichilemmal cysts frequently present in an autosomal dominant pattern^5^ but can also appear sporadically. Patients with hereditary trichilemmal cysts typically develop multiple lesions that start at an earlier age than those with sporadic lesions^6^. In an attempt to localize the gene mediating autosomal dominant inheritance of trichilemmal cysts, Eiberg and coworkers^7^ applied genome wide linkage analysis to a large Danish family. They identified a region on chromosome 3p24-p21.2 that likely contained the causative gene for inheritance of trichilemmal cysts and termed this locus TRICY1. Further attempts by this group and others to identify the specific gene were unsuccessful.

We initially hypothesized that trichilemmal cysts represent clonal proliferations and further conjectured that the familial presentation of these lesions was an example of an autosomal dominant tumor syndrome explained by Knudson’s two-hit hypothesis^8^. According to this model, both alleles of a tumor suppressor gene must be knocked out for a tumor to form. The function of one copy is partially or fully lost due to an inherited genetic variation or mutation. Thus, this model predicts that a tumor subsequently develops after a cell loses the tumor suppressing activity of the remaining copy through somatic mutation. Alternatively, two hits to the same allele could be required to produce the phenotype. We applied whole exome sequencing (WES) and Sanger sequencing to identify germline variants and somatic mutations that potentially underlie these cysts and subsequently determined if the two hits were on the same or opposite alleles.

## Results

We filtered WES data from the discovery cohort for genes mutated in more than half of the cysts when compared to the corresponding blood sequence. Our screen produced only one gene, phospholipase C delta 1 (*PLCD1*). Remarkably, all 15 trichilemmal cysts harbored the exact same p.S745L (c.2234 G > A), hg38 chr3:38,007,810) somatic mutation in exon 15, resulting in an amino acid change from serine to leucine (Table 1). We confirmed this finding by WES of six additional familial FFPE trichilemmal cysts from four unrelated individuals (without matching blood). All six of these additional specimens showed the p.S745L mutation. Thus, as shown in Table 2, all 21 familial cysts that underwent WES harbored the p.S745L somatic mutation. This somatic mutation was present in approximately half of the reads from each cyst except for two of the FFPE cysts that exhibited infiltration by inflammatory cells (data not shown). Thus, samples containing nearly pure cyst were heterozygous for the p.S745L somatic mutation, indicating that trichilemmal cysts are clonal tumors.

**Table 1:** *PLCD1* Germline and Somatic Mutations/Variants in Familial Trichilemmal Cysts.

Fresh Tissue with Paired Blood			
		Germline Mutations/Variant	Somatic Mutations
Patient 1	Blood	p.S460L (c.1376G > A)	
	Cyst A		p.S745L (c.2234G > A)
	Cyst B		p.S745L (c.2234G > A)
Patient 2	Blood	p.S460L (c.1379G > A)	
	Cyst A		p.S745L (c.2234G > A)
Patient 3	Blood	p.S460L (c.1379G > A)	
	Cyst A		p.S745L (c.2234G > A)
	Cyst B		p.S745L (c.2234G > A)
Patient 4	Blood	p.S460L (c.1379G > A)	
	Cyst A		p.S745L (c.2234G > A)
Patient 5	Blood	pE455K (c.1363C > T)	
	Cyst A		p.S745L (c.2234G > A)
	Cyst B		p.S745L (c.2234G > A)
	Cyst C		p.S745L (c.2234G > A)
	Cyst D		p.S745L (c.2234G > A)
Patient 6	Blood	p.S460L (c.1379G > A)	
	Cyst A		p.S745L (c.2234G > A)
	Cyst B		p.S745L (c.2234G > A)
	Cyst C		p.S745L (c.2234G > A)
	Cyst D		p.S745L (c.2234G > A)
	Cyst E		p.S745L (c.2234G > A)
**FFPE Tissue Without Blood**			
		**Germline Mutations/Variant**	**Somatic Mutations**
Patient 7	Cyst A	p.S460L (c.1379G > A)	p.S745L (c.2234G > A)
Patient 8	Cyst A	p.S460L (c.1379G > A)	p.S745L (c.2234G > A)
	Cyst B		p.S745L (c.2234G > A)
Patient 9	Cyst A	p.S460L (c.1379G > A)	p.S745L (c.2234G > A)
	Cyst B		p.S745L (c.2234G > A)
Patient 10	Cyst A	p.S460L (c.1379G > A)	p.S745L (c.2234G > A)

**Table 2:** *PLCD1* variants/mutations in Patients with Familial Trichilemmal Cysts.

Mutation/Variant	Frequency in Population	Frequency of Cohort
Germline: p.S460L (c.1379 G > A)	6.00% (European)	16/17 pts (94%)*
Germline: p.E455K (c.1363C > T)	0.04%	1/17 pts (6%)
Somatic: p.S745L (c. 2234G > A)	N/A	21/21 cysts (100%)
*P value = 4.5 × 10^−19^		

Since the tendency to form trichilemmal cysts is inherited in an autosomal dominant manner, we manually searched all exons of *PLCD1* for germline variants that might provide the first “hit”. Nine of the ten patients that underwent WES were heterozygous for the p.S460L (c.1379 G > A, hg38 chr3:38,009,720, rs75495843) germline variant in exon nine, also resulting in an amino acid change from serine to leucine. This variant is present in about 6% of all individuals of European and South Asian ancestry and is less common in persons of other genetic backgrounds. No other germline variants in *PLCD1* were identified in more than one subject. To further validate this finding, we performed Sanger sequencing of *PLCD1* exon nine amplicons from seven additional individuals with multiple trichilemmal cysts. All seven were heterozygous for the p.S460L variant. Thus, as shown in Table 2, 16/17 individuals with multiple trichilemmal cysts showed the p.S460L variant (p = 4.5 × 10^−19^). The 1/17 that did not show this variant harbored a rare *PLCD1* germline mutation in exon nine, p.E455K (c.1363 C > T, hg38 chr3:38,009,736, rs141555869) resulting in an amino acid change from glutamic acid to lysine, present in about 0.04% of humans^9^.

We next tested whether the inherited variant and the somatic mutation occurred on the same chromosome (in cis) or on opposite chromosomes (in trans). A 2.7 KB sequence containing both exons nine and fifteen was amplified from genomic DNA purified from five cysts. For each cyst, ten bacterial colonies containing the 2.7 KB amplicon TA cloned into a plasmid were analyzed for both the c.2457 G > A somatic mutation and the c.1379 G > A germline variant using Sanger sequencing. As shown in Table 3, the c.2457 G > A somatic mutation was always found on the same amplicon as the c.1379 > A germline variant (p = 0.03). There were a few reads that showed only the c.1379 G > A germline variant which likely represent inflammatory cells contaminating the cyst. Our results indicate that familial trichilemmal cysts result from a combination of germline and somatic mutations on the same copy of the *PLCD1* gene.

**Table 3:** *PLCD1* Mutations/Variants in Single Colony Sequences from Familial Trichilemmal Cysts.

Number of Amplicons with Indicated Genotype				
Subject/Cyst	1379G/2234G	1379G > A/2234G > A	1379G/2234G > A	1379G > A/2234G
**Subject 1**				
Cyst 1	4	4	0	2
Cyst 2	3	6	0	1
**Subject 2**				
Cyst 1	3	6	0	1
**Subject 3**				
Cyst 1	2	3	0	5
**Subject 4**				
Cyst 1	2	7	0	1

Given that our cohort with familial trichilemmal cysts all harbored germline variants or mutations in exon 9 and somatic mutations in exon 15 of *PLCD1*, we asked whether apparently sporadic trichilemmal cysts were a distinct genetic entity or merely the result of incomplete penetrance. We performed Sanger sequencing of lesional tissue and blood in a cohort of subjects with apparently sporadic trichilemmal cysts. *PLCD1* exon 9 was sequenced in all of these subjects while *PLCD1* exon 15 was sequenced in trichilemmal cysts that were greater than 75% pure.

Of these 15 subjects with apparently sporadic trichilemmal cysts, six were heterozygous for the p.S460L variant (Table 4) (p = 1.3 × 10^−4^) suggesting that six of the 15 individuals actually had familial cysts with incomplete penetrance or inaccurate family histories. All six of these subjects exhibited the p.S745L somatic mutation in exon 15 of *PLCD1* (data not shown). The remaining nine of the15 subjects appeared to exhibit truly sporadic trichilemmal cysts. As shown in Table 5, these subjects exhibited no germline variants or mutations in exon 9 or exon 15 of *PLCD1*. Interestingly, only half of these cysts showed the p.S745L somatic mutation in exon 15 of *PLCD1* as compared to 100% of the familial trichilemmal cysts.

**Table 4:** *PLCD1* variants/mutations in patients with Single Trichilemmal Cyst, Denies Family History.

Mutation/Variant	Frequency in Population	Frequency in Cohort
Germline: p.S460L (c.1379G > A)	6.00% (European)	6/15 pts (40%)*
Germline: p.E455K (c.1363C > T)	0.04%	0/15 pts (0%)
Somatic: p.S745L (c.2234G > A)	N/A	8/13† cysts (62%)

*P value = 1.3 × 10^−4^.

^†^Subjects with <75% tumor in specimen were exclueded.

**Table 5:** *PLCD1* variants/mutations Patients with a Single Trichilemmal Cyst, w/o p.S460L mutation.

Mutation/Variant	Frequency in Population	Frequency in Cohort
Germline: p.S460L (c.1379G > A)	6.00% (European)	0/9 pts (0%)
Germline: p.E455K (c.1363C > T)	0.04%	0/9 pts (0%)
Somatic: p.S745L (c.2234G > A)	N/A	3/6† cysts (50%)

^†^Subjects with <75% tumor in specimen were exclueded.

As a negative control, we sequenced epidermal inclusion cysts from five subjects (of European genetic background) without any history of trichilemmal cysts. We performed WES on lesional tissue and blood from two subjects and Sanger sequencing of *PLCD1* exons 9 and 15 on the remaining three cysts. None of these individuals demonstrated germline or lesional mutations in *PLCD1* (Table 6).

**Table 6:** *PLCD1* variants/mutations Patients with Epidermal Inclusion Cysts.

Mutation/Variant	Frequency in Population	Frequency in Cohort
Germline: p.S460L (c.1379G > A)	6.00% (European)	0/5 pts (0%)
Germline: p.E455K (c.1363C > T)	0.04%	0/5 pts (0%)
Somatic: p.S745L (c.2234G > A)	N/A	0/5 cysts (0%)

## Discussion

We present clear evidence that a combination of an inherited *PLCD1* variant, p.S460L, with a subsequent p.S745L mutation in the same gene (on the same chromosome) causes multiple trichilemmal cysts. *PLCD1* is a member of the phospholipase C family of enzymes that hydrolyzes phosphatidylinositol 4,5-bisphosphate to produce the second messenger inositol 1,4,5-trisphosphate (IP3) and diacylglycerol (DAG). IP3 is a key regulator of intracellular Ca^++^ release^10^, while DAG activates protein kinase C. *PLCD1* is expressed at high levels in most tissues including the skin. *PLCD1* knockout mice show abnormal hair formation, increased cutaneous inflammation and, most relevant to this study, follicular cysts^11,12^. Various rare germline *PLCD1* mutations are associated with both autosomal dominant and autosomal recessive leukonychia in humans^13,14^. However, the *PLCD1* variants causing leukonychia are distinct from those identified in this study. Interestingly, the autosomal dominant transmission of both trichilemmal cysts and leukonychia has been observed in several families but no genetic data is available for this syndrome^15^.

Ultraviolet (UV) radiation characteristically alters DNA by producing CC to TT or C to T base substitutions at dipyrimidinic sites. The p.745 L mutation results in a nucleotide change of TCA to TTA which is consistent with a UV signature. However, further study will be required to determine if UV exposure actually increases the risk for trichilemmal cysts. The p.S745L mutation is absent from the COSMIC database of currently known cancer mutations^16^.

Both the common inherited variant and the somatic mutation eliminate distinct serine phosphorylation sites on *PLCD1*^17^. Moreover, p.S745, p.S460 and p.E455 are all conserved in mammals (M. musculus), birds (G. gallus) and amphibians (X. tropicalis). PolyPhen-2 is a tool that predicts the functional effects of amino acid changes via analysis of sequence alignments and protein structures^18^. We used this tool to determine if the mutations/variants identified in *PLCD1* would likely disrupt enzyme function. p.S745L, p.S460L and p.K455E were all predicted to be “probably damaging” with scores of 1.0/1.0, 0.998/1.0 and 0.989/1.0 respectively.

Our results partially overlap with a very recently published study by Horer and coworkers. Both studies identified the same novel single allele, two hit mechanism for hereditary trichilemmal cysts. However, there were some differences between the two studies. The Horer^19^ study included only five patients with apparently sporadic trichilemmal cysts and all five of these subjects carried the p.S460L germline risk allele. In contrast, our study included 15 subjects with apparently sporadic cysts and only 40% of these subjects harbored the risk allele indicating the risk allele is not required for formation of non-syndromic trichilemmal cysts. These differences may represent different genetic populations or the greater number of sporadic cysts in our study. We also exome sequenced numerous separate cysts originating from the same individual and found the identical somatic mutation in each cyst. Moreover, we identified a distinct high-risk variant, p.E455K that was present in one of our subjects instead of the p.S460L allele.

Our findings, along with those of Horer, show that the germline variant and the somatic mutation were on the same allele of *PLCD1* was surprising as it is inconsistent with the currently accepted model for tumor syndromes caused by tumor suppressor genes (Knudson’s two hit hypothesis). Mouse studies suggest the loss of PLCD1 function can promote trichilemmal cyst formation. Approximately equal copies of both alleles were present in all specimens that were nearly pure tumor. Thus, we think loss of heterozygosity is an unlikely explanation why loss of function in only one allele can result in a tumor. Since we have not performed functional studies, we can only speculate on the mechanism that the genetic changes result in the trichilemmal cyst phenotype. However, Horer and coworkers^19^ have shown that the somatic and germline mutations cause additive loss in *PLCD1* enzyme activity. Thus, somatic and germline mutations in the same allele may combine to cause loss of function thereby reducing the amount of active enzyme. Additionally, a dominant negative protein produced by the mutant allele could inhibit function of the wildtype enyme. Clearly, further research is needed to determine how the two genetic changes on the same copy of the gene can act cooperatively to produce a cyst in the presence of functional protein produced from the opposite allele.

The c.1379 C > T germline variant, found in 16/17 subjects, is seen in only 6% of people with European or South Asian ancestry and is less common in other populations9. Since all of our subjects were of European ancestry, we would have expected to see only one of 17 subjects with the c.1379 C > T variant if this variant was not linked to trichilemmal cysts. Instead, we found the c.1379 C > T variant in 94% (16/17) of our patients with familial trichilemmal cysts. The one patient without c.1379 C > T had a rare mutation in the same exon of *PLCD1* not seen in any of the other subjects suggesting that this mutation may have functional sequelae similar to c.1379 C > T. Our finding that germline *PLCD1* variants cause familial trichilemmal cysts is further supported by the localization of *PLCD1* within the larger genetic region previously associated with the formation of hereditary trichilemmal cysts in a single family^7^.

In summary, we have used WES to identify a mutation in *PLCD1* (p.S745L) that is present in 100% of familial trichilemmal cysts. This somatic mutation exhibits a UV signature suggesting that sun protection of the scalp may prevent these lesions. Moreover, we have shown that inheritance of trichilemmal cysts is mainly due to single nucleotide polymorphisms (SNPs) in exon nine of the *PLCD1* gene, predominantly c.1379 C > T. This SNP has a prevalence in the US population similar to that of trichilemmal cysts.

Although the origin of common cutaneous cysts has remained unclear, less common cysts have received more attention at the genetic level Like trichilemmal cysts, steatocystomas present both as solitary lesions and as a genetic syndrome inherited through activating mutations in the keratin 17 (K17) gene^20^. Pilomatrixomas are associated with somatic mutations in CTNNB1, which codes for Beta-Catenin, resulting in activation of WNT signaling^21^. EICs are often conceptualized as hamartomas that result from injured hair follicles or from keratinocytes traumatically implanted into the dermis rather than as clonal tumors^22^. Our findings demonstrate that trichilemmal cysts are clonal tumors and the familial presentation of these lesions is an autosomal dominant tumor syndrome. Clinicians treating these patients can now clearly explain the nature of this disorder to affected families and suggest potential actions that might prevent future cysts. Future studies are needed to determine if this tumor syndrome consists exclusively of benign cysts or is also associated with other neoplasms.

### Limitations

Our study only included subjects of European genetic background living in Virginia, West Virginia and Pennsylvania. Persons of different genetic background may have distinct germline variants and somatic mutations. Moreover, persons with more pigmented skin types may exhibit different somatic mutations due to decreased penetrance of UV radiation.

## Methods

### Samples

Our cross-sectional study included two cohorts of familial trichilemmal cysts and one cohort of apparently sporadic trichilemmal cysts. For our first (discovery) cohort, we invited all patients who came to our clinic for trichilemmal cyst removal and had evidence of multiple lesions (either presented with multiple trichilemmal cysts at the time of the visit or based on the medical record) to participate in the study. We then excluded any patient that denied having a first degree relative with trichilemmal cysts. For this cohort, we conducted WES on^15^ fresh frozen trichilemmal cysts (with matched blood) from six individuals. To determine if the common germline variant and the somatic mutation occurred on the same or opposite alleles, we obtained five additional trichilemmal cyst from four individuals. We PCR amplified a 2.7 KB sequence from each cyst spanning both exon 9 and 15 of *PLCD1* and performed TA cloning, single colony Sanger sequencing and as described in the supplemental methods.

Our second cohort consisted of formalin-fixed, paraffin-embedded (FFPE) trichilemmal cysts (without matched blood) from 11 individuals. These subjects were selected using our electronic medical records database (EMR) system to identify persons with multiple trichilemmal cysts. We then performed WES on six cysts from the four most recent individuals. Next, we sequenced PCR amplicons of *PLCD1* exon nine from seven cysts from the seven remaining subjects.

Our third cohort of apparently sporadic trichilemmal cyst subjects was selected by screening the EMR at our institution for patients with single trichilemmal cysts. Potential subjects were contacted by phone and questioned about any history of other scalp cysts or nodules and any family history of these lesions. These paraffin embedded cyst subjects were chosen from the most recent subjects in our EMR database that denied multiple cysts and familial history. Two of the paraffin embedded cysts for WES and two of the cysts in the apparently sporadic cohort exhibited inflammatory cells indicating possible rupture. Five of the 15 subjects with apparently sporadic trichilemmal cysts provided blood or other systemic DNA. We sequenced exons nine and 15 as indicated in the supplementary methods. To our knowledge, all subjects were unrelated. This study was approved by the West Virginia University Blue Institutional Review Board (Morgantown, WV) and the Carilion Institutional Review Board (Roanoke, VA). All research was performed in accordance with relevant guidelines/regulations. Informed consent was obtained from each subject. Statistics were calculated using a binomial probability model.

### WES

Exome capture was performed using Agilent SureSelect Human All Exon UTR (v5) kit according to manufacturer’s protocol. WES sequencing was carried out with the Illumina Hiseq. 125 cycle paired end sequencing v4 Protocol. A mean coverage of 93x and 88x was obtained for cyst and blood, respectively.

### Sanger Sequencing

DNA was extracted from FFPE Tissues using the Qiagen QIAamp DNA FFPE Tissue protocol. Genomic DNA was used as template to amplify exon 9 of *PLCD1* with AmpilTaq Gold DNA Polymerase (Thermo Fisher). Forward Primer, GCTGTTGAACCGACCGACCACTG and reverse primer was CAGGCGCAGAGAGACAGAG. For exon 15 of *PLCD1*, forward primer was tccctTGAyGCATCCACCCC and reverse primer was agccytgtccccacaTGTGG. The purified PCR products were directly sequenced using the BigDye® Terminator v3.1 Cycle Sequencing Kit (Applied Biosystems).

### TA cloning of PCR amplicons

A 2.7 KB sequence was amplified from genomic DNA isolated from each cyst using the following primers. Forward TGGAGGCAGCGGCAGGAGAAAAAG, reverse CCCCATGCTGTTGAACCGACCAC. TA cloning and Sanger sequencing was performed by Genewiz (South Plainfield, NJ).

### Bioinformatics

Somatic mutations were identified and screened by the Sention TNseq Algorithm on the DNAnexus platform. Integrative Genomics Viewer (Broad Institute) was used for visualization of the sequence data in comparison to hg38.
